# Socio-Demographic Patterning of Physical Activity across Migrant Groups in India: Results from the Indian Migration Study

**DOI:** 10.1371/journal.pone.0024898

**Published:** 2011-10-14

**Authors:** Ruth Sullivan, Sanjay Kinra, Ulf Ekelund, Bharathi A.V., Mario Vaz, Anura Kurpad, Tim Collier, Kolli Srinath Reddy, Dorairaj Prabhakaran, Yoav Ben-Shlomo, George Davey Smith, Shah Ebrahim, Hannah Kuper

**Affiliations:** 1 Department of Non-communicable Disease Epidemiology, London School of Hygiene and Tropical Medicine, London, United Kingdom; 2 Medical Research Council, Epidemiology Unit, Cambridge, United Kingdom; 3 Indira Gandhi National Open University, Bangalore, India; 4 St. John's Research Institute, Bangalore, India; 5 Department of Medical Statistics, London School of Hygiene and Tropical Medicine, London, United Kingdom; 6 Public Health Foundation of India, New Delhi, India; 7 Centre for Chronic Disease Control, New Delhi, India; 8 School of Social and Community Medicine, University of Bristol, Bristol, United Kingdom; 9 Medical Research Council Centre for Causal Analyses in Translational Epidemiology, University of Bristol, Bristol, United Kingdom; 10 South Asia Network for Chronic Disease, Public Health Foundation of India, New Delhi, India; Lerner Research Institute, Cleveland Clinic, United States of America

## Abstract

**Objective:**

To investigate the relationship between rural to urban migration and physical activity (PA) in India.

**Methods:**

6,447 (42% women) participants comprising 2077 rural, 2,094 migrants and 2,276 urban were recruited. Total activity (MET hr/day), activity intensity (min/day), PA Level (PAL) television viewing and sleeping (min/day) were estimated and associations with migrant status examined, adjusting for the sib-pair design, age, site, occupation, education, and socio-economic position (SEP).

**Results:**

Total activity was highest in rural men whereas migrant and urban men had broadly similar activity levels (p<0.001). Women showed similar patterns, but slightly lower levels of total activity. Sedentary behaviour and television viewing were lower in rural residents and similar in migrant and urban groups. Sleep duration was highest in the rural group and lowest in urban non-migrants. Migrant men had considerably lower odds of being in the highest quartile of total activity than rural men, a finding that persisted after adjustment for age, SEP and education (OR 0.53, 95% CI 0.37, 0.74). For women, odds ratios attenuated and associations were removed after adjusting for age, SEP and education.

**Conclusion:**

Our findings suggest that migrants have already acquired PA levels that closely resemble long-term urban residents. Effective public health interventions to increase PA are needed.

## Introduction

India, like other low and middle income countries (LMIC), is in a state of transition with marked social, demographic and epidemiological changes underway [1]. This shift is characterised by increased longevity underlying population growth; an increasingly urbanised population, projected to rise from 28% in 2001 to 50% by 2025 [2] and a rising burden of chronic disease. In 2005 around 53% of India's mortality was attributed to chronic disease [3], a figure that is estimated to reach approximately 68% (8 million deaths) by 2020 [2], [3]. As urbanisation continues with the expansion of cities and rural-urban migration, so too has the adoption of a more urbanised lifestyle, characterised by lower levels of physical activity (PA) and a diet higher in saturated fats [4], [5], [6], [7]. These behavioural shifts have contributed to the increase in cardiovascular disease (CVD) risk factors such as obesity, diabetes, and hypertension [6], [8], [9].

PA is a well-established modifiable risk factor for chronic disease [10], [11], with over 1.9 million deaths globally a year attributable to inactivity [12]. Health benefits associated with PA include lowered blood pressure, reduced body fat and central adiposity, enhanced musculoskeletal health and improved glucose metabolism, in turn reducing the risk of CVD, obesity, diabetes and certain cancers [13], [14].

PA is a complex exposure due to its multi-dimensional nature [15]. Activity participation is usually categorised by duration of time spent active and by time spent in different levels of activity intensity; (sedentary, light, moderate and vigorous). Evidence suggests that maximum health benefits occur when activity is of at least moderate intensity (e.g. walking briskly, vacuuming, cycling) [16], and in bouts of at least 10 minutes duration. There is evidence that prolonged periods of sedentary activity such as sitting and television viewing may be independent risk factors for chronic disease [17].

Across India chronic disease levels vary markedly, with lower levels of obesity and diabetes in rural areas and amongst men [18], with the prevalence peaking in higher socio-economic groups [19]. Inactivity is a risk factor for obesity, CVD and diabetes [20], and it is possible that the distribution of these health conditions seen within India, may partly be attributed to differing activity participation. Using data on 6,447 participants from the Indian Migration Study (IMS) [18], [21], which aimed to investigate the effects of rural to urban migration on obesity and diabetes, this study of PA represents the largest of its kind in India. We quantify the pattern of PA within India by migrant status, to establish whether the PA transition (declining PA levels and increasing sedentary behaviour) [22] is underway in both urban and rural areas. We hypothesised that PA patterns would show an inverse gradient from rural non-migrants, to rural-urban migrants and urban non-migrants.

## Methods

### Study Population

The design of the IMS has been described previously [18], [21]. In summary, the IMS is nested within a cardiovascular disease surveillance project [23] of industrial populations in four large cities from the north, centre and south of India (Lucknow, Hindustan Aeronautics Ltd; Nagpur, Indorama Synthetics Ltd; Hyderabad, Bharat Heavy Electricals Ltd; and Bangalore, Hindustan Machine Tools Ltd). Factory workers and their co-resident spouses were asked to provide information on rural-to-urban migration and family status. Those responding positively, along with a 25% random sample of urban non-migrants, were asked to participate in the study. Each participant was asked to identify one non-migrant full sibling, preferably of the same sex and closest in age to them, who was invited to participate in the study. If no full-sibling was available a cousin or close friend from the same village was invited. The field work for the study ran from March 2005 to December 2007.

### Measurements

All participants completed an interviewer-administered questionnaire to gather information on socio-demographic and lifestyle data. A quantitative physical activity questionnaire (IMS-PAQ) specific to the Indian population was developed and validated to gather information on a participant's habitual daily activity over the last one month [24], [25]. Participants were asked to recall information about main activity domains; sleep, occupation (sitting, standing, walking and activities more strenuous than walking at work), exercise, household, hobby, sedentary and other (e.g. eating, dressing, travelling to and from work). The hobby category was later dropped from further analyses and its information reallocated to appropriate domains as activities reported were predominantly household chores rather than true hobbies or pastimes. The IMS-PAQ asked primarily open-ended questions and participants reported up to 21 activities specific to them, rather than responding to a set list of activities e.g. ‘*apart from work, how do you spend your time (over last one month): i) Sports/games/exercise (for example walking, badminton, jogging, cricket….etc).*’ The exceptions to this were for sleep, standing, sitting, walking at work, and ‘other’ activities which were closed-questions, e.g. ‘*on average how many hours in a day do you sleep?*’ For each activity reported, additional information was gathered on its frequency and duration. Duration was reported in minutes per day and frequency within 6 categories (Daily; Once a week; 2–4 times a week; 5–6 times a week; once a month; 2–3 times a month).

Trained personnel took anthropometric measures of height and weight from all participants during the clinic visit. Height was measured to the nearest 0.1 cm using a portable stadiometer with a base plate (Leicester height measure, Chasmore Ltd. London). Weight was measured twice, to the nearest 0.1 kg using a digital scale (Model PS16, Beurer, Germany), with participants removing their shoes and wearing light clothing. The average of measures taken was used for these analyses. All protocols and equipment were pilot tested prior to the study commencing. Fieldworkers at the four study sites underwent joint training sessions and standardisation at the outset and subsequently every six months. Anthropometric instruments were calibrated at the start of each clinic session.

### Statistical analysis

All statistical analyses were conducted using STATA software version 11. Education was classified as: no formal education; primary (1 to ≤4 years); secondary (5 to ≤12 years), and beyond secondary. Occupation was classified as housework, unemployed, manual, skilled manual and non-manual/professional. Body mass index (BMI, kg/m^2^) was classified using the Asian (<18.5 kg/m^2^; 18.5 to 23.0 kg/m^2^; >23.0 to 25.0 kg/m^2^; >25.0 kg/m^2^) cut-points [26]. The Standard of Living Index (SLI) was calculated by applying standard weights to subsets of questions, and rescaling them to the full score [19]. The score was then categorised into tertiles to produce low, medium and high socio-economic position (SEP) groups.

Physical activities reported within the IMS were assigned a metabolic equivalent value (MET) using the Compendium of Physical Activity and WHO/FAO/UN guidelines, supplemented with country specific values [16], [27], [28]. One MET is equivalent approximately 3.5 mL of O_2_/kg/min, or 1 kcal/kg/hour, corresponding to the resting metabolic rate of sitting quietly [16]. The following physical outcome measures were generated:

1. Time spent on each activity was calculated as minutes/day using information recalled on activity duration and frequency. Individual activity durations were summed to generate total daily duration of recalled activities. If this value was less than 24 hours, a residual time variable was generated and a standard MET value of 1.4 applied as in previous studies [24], [29]. Participants over-reporting time spent in daily activities (i.e. >24 hours) had the duration of each individual activity reduced proportional to the amount over-reported.
2. Total activity was calculated as Total MET (hr/day) by summing daily MET values of all activities. For occupational activity ‘more strenuous than walking’ the Integrated Energy Index (IEI) was applied [30], to correct total MET. This adjusts for unreported rests which occur when participants report strenuous occupational activities such as digging, which are too physically demanding to occur for prolonged periods without short breaks. Four categories of total activity (MET hr/ay) were subsequently created using quartile cut-points for men and women separately.
3. Time (total time [min/day]), spent in categories of activity intensity were generated using previously published cut-points; sedentary <1.5 MET; light 1.5 to 3 MET; moderate 3 to 6 MET; vigorous >6 METs [31]. Time spent in moderate and vigorous activity was subsequently combined (MVPA) as only 3% of the sample reported activity of a vigorous intensity.
4. A Physical Activity Level (PAL) was calculated (estimated 24 hour energy expenditure divided by predicted basal metabolic rate [BMR] using the Schofield equation) [27], [32]. Previously established cut-points were then applied to the PAL value to group individuals into one of four categories: PAL <1.40 *extremely inactive lifestyle*, PAL 1.40–1.69 *sedentary/lightly active lifestyle*, PAL 1.70–1.99 *moderately active lifestyle*, PAL≥2.0 *vigorously active lifestyle* [27].

Linear regression was applied to assess differences in PA outcomes between migrant and socio-demographic groups, adjusting for age, sex and factory site. We then repeated these analyses but stratified by sex and migrant status and additionally estimated the proportion of participants (95%, Confidence Interval [CI]) within PAL categories. Data for MVPA and television viewing which were positively skewed are presented as geometric mean (95% CI), and were log-transformed prior to regression analyses. To test for interactions between migrant status and sex on PA outcomes linear regression was applied.

To investigate how much of the difference between migrant groups could be explained by socio-demographic factors, logistic regression was applied. Categories of total activity (MET hr/day) were used as the exposure, initially comparing migrant and rural participants, then migrant and urban participants, stratified by sex and using rural and urban participants respectively as baseline. Results are presented as odds ratios (OR), initially unadjusted, and subsequently adjusting for age, factory site and socio-demographic variables. P-values were calculated for association and trend (linear and non-linear by generating a quadratic exposure variable).

All analyses were adjusted, where applicable, with robust standard errors to account for the clustered nature of the data (sibling-pairs). Wald tests were performed on model parameters.

### Ethics Approval

Information sheets were translated into local languages and signed (or a witnessed thumb print obtained if the participant was illiterate), and through this, informed consent was obtained. Ethics committee approval (including this process for obtaining informed consent) was obtained from the All India Institute of Medical Sciences Ethics Committee, reference number A-60/4/8/2004 and the London School of Hygiene and Tropical Medicine.

## Results

### Response Rates

A total of 13,695 participants completed an assessment of their eligibility for the study of whom 7,594 (55%) fulfilled the inclusion criteria as they had a rural dwelling sibling or were part of a random 25% sample of urban non migrants [18]. Of the 7,102 (94%) who agreed to complete the clinical examination with their sibling, a total of 3,537 (50%) sibling-pairs attended the clinic. Six hundred and twenty seven individuals (9%) were excluded from these analyses; 519 migrated to work from rural areas, 38 were urban-rural migrants (so not in the migrant categories of interest), seven had incomplete questionnaire data, 62 accounted for ≤12 hours of activity daily and one reported ≥36 hours of activity. This left a total of 6,447 participants for the present analysis on whom complete data was available except for PAL (3 missing).

### Socio-demographic characteristics

Table 1 presents descriptive characteristics of the study population. The sample comprises 6,447 adults aged 17–76 years (mean 41.0 years, [SD 10.2]); 32% were rural participants and 42% were women. Education levels were lower in rural participants, particularly amongst women, 34% of whom had no formal education compared to 3% of urban women. Non-manual/professional work was more common in urban participants (48% urban men vs. 22% rural men; 17% urban women vs. 8% rural women). Socio-economic position was lowest in rural men and women (68% rural, 16% migrant, 18% urban residents categorised into the lowest tertile using the Standard of Living scores). Obesity levels (BMI>25 kg/m^2^) were higher in urban than rural areas (urban women 54% vs. 28% rural women; urban men 41% vs. 18% rural men) and higher in women than men (47% vs. 33%).

**Table 1:** Characteristics of the Indian Migration Study participants.

	Men (n = 3,768)			Women (n = 2,679)		
	Rural (1,443)	Migrant (1,127)	Urban (1,198)	Rural (634)	Migrant (967)	Urban (1,078)
**Age group (%)**						
<30	21.6	3.4	12.5	15.8	17.0	17.3
30–39	27.9	24.9	27.4	25.5	25.5	30.1
40–49	30.2	36.9	36.8	31.3	44.5	36.9
≥50	20.4	35.9	23.3	27.4	13.0	15.8
**BMI Categories (%)**						
<18.5 kg/m^2^	22.3	3.4	6.2	18.2	7.5	8.4
18.5–<23 kg/m^2^	45.4	32.0	30.0	39.0	24.0	3.6
23.0–<25.0 kg/m^2^	14.1	23.9	21.6	14.7	16.9	14.5
≥25 kg/m^2^	18.2	40.8	42.2	28.1	51.7	53.5
**Education (%)**						
No formal education	11.9	1.1	1.2	34.3	19.7	3.3
Up to primary (≤4 years)	15.8	3.7	5.7	26.6	24.5	9.3
Up to secondary (≤12 years)	50.8	65.0	47.1	29.3	43.1	43.8
Beyond secondary	21.6	30.2	46.1	9.8	12.7	43.7
**Occupation (%)**						
Housework	1.1	0.4	1.6	66.4	89.5	69.4
Unemployed	8.2	0.6	7.3	7.3	0.1	3.4
Manual	50.6	3.7	11.9	15.1	2.1	2.9
Skilled manual	18.5	56.6	31.6	3.3	2.9	7.1
Non-manual/Professional	21.6	38.6	47.6	7.9	5.5	17.3
**Standard of Living Index (%)** a						
Low	66.8	15.4	19.4	69.9	17.3	17.4
Middle	23.7	44.5	45.4	23.0	48.7	45.4
High	9.5	40.0	35.2	7.1	34.0	37.2
**Geographical region (%)** b						
North	55.8	51.1	59.1	36.2	47.5	52.3
South	44.2	48.9	40.9	63.8	52.5	47.0

Data presented are frequency proportions (%).

a Based on a subset of questions from the Standard of Living Index. Scores are based on tertiles.

b South includes the four southern states of Andhra Pradesh, Kerala, Karnataka and Tamil Nadu.

### Physical activity characteristics

PA characteristics for the sample as a whole are shown in table 2. Overall, mean PAL was 1.62 (SD, 0.19), equivalent to a *sedentary/lightly active lifestyle*, although substantial variation existed across socio-demographic groups. Considerably more time was reported in sedentary behaviour (mean 475 min/day [SD 163]) compared to MVPA (geometric mean 95 min/day [95% CI 92, 98]).

**Table 2:** Distribution of Physical Activity characteristic (mean, standard deviation [SD])^1^ of the Indian Migration Study (n = 6,447).

		Total Activity (MET hr/day)	Physical Activity Level (PAL)†	Time Spent in Activities (min/day)				
	N			Activity Intensity¥			Television Viewing & Sleeping	
				Sedentary	Light	Moderate or Vigorous	Television Viewing	Sleeping
**Total**	6,447	38.8 (4.5)	1.62 (0.19)	475 (163)	370 (133)	95 (92–98)	35 (34–37)	442 (63)
**Age Group (years)**								
<30	949	39.3 (5.1)	1.64 (0.21)	466 (162)	366 (139)	98 (91–106)	36 (32–41)	449 (67)
30–39	1,732	39.5 (4.6)	1.64 (0.19)	452 (161)	384(134)	102 (96–108)	32 (29–35)	441 (63)
40–49	2,319	38.6 (4.3)	1.61 (0.18)	481 (160)	373(131)	89 (85–94)	40 (37–43)	442 (62)
≥50	1,447	38.1 (4.4)	1.59 (0.18)	498 (168)	351(129)	93 (86–99)	33 (30–36)	442 (64)
p-value age^a^		<0.001	<0.001	<0.001	0.021	0.509	0.652	<0.001
**Sex**								
Men	3,768	39.5 (4.8)	1.65 (0.20)	475 (165)	334 (121)	147 (142–151))	30 (28–32)	435 (62)
Women	2,679	37.8 (4.0)	1.58 (0.17)	474 (161)	420 (132)	52 (48–54)	45 (42–48)	454 (63)
p-value sex^a^		<0.001	<0.001	0.622	<0.001	<0.001	<0.001	<0.001
**Migrant Status**								
Rural	2,077	40.5 (5.4)	1.69 (0.22)	421(175)	355 (130)	142 (135–150)	15 (14–17)	450 (64)
Migrant	2,094	37.9 (3.7)	1.58 (0.15)	491 (145)	384 (129)	77 (73–81)	50 (46–53)	442 (61)
Urban	2,276	38.0 (3.9)	1.59 (0.16)	508 (155)	371 (138)	79 (75–83)	55 (53–59)	436 (64)
p-value urban/rural^a^		<0.001	<0.001	<0.001	0.748	<0.001	<0.001	<0.001
**Socio-Economic Position** b								
Low SEP	2,168	40.4 (5.2)	1.68 (0.22)	423 (172)	368 (134)	132 (126–139)	17 (15–18)	448 (66)
Medium SEP	2,494	38.2 (4.1)	1.59 (0.17)	494 (155)	372 (134)	83 (79–87)	49 (46–53)	440 (64)
High SEP	1,785	37.7 (3.6)	1.57 (0.15)	509 (147)	370 (131)	76 (71–80)	56 (52–59)	439 (59)
p-value SEP^a^		<0.001	<0.001	<0.001	0.111	<0.001	<0.001	<0.001
**Geographic region** c								
North	3,349	39.3 (4.6)	1.64 (0.19)	467 (156)	372 (125)	106 (102–111)	32 (30–34)	438 (62)
South	3,098	38.3 (4.6)	1.60 (0.19)	483 (170)	368 (141)	83 (79–88)	40 (37–43)	447 (64)
p-value north/south^a^		<0.001	<0.001	0.075	<0.001	0.595	<0.001	<0.001

1 Data presented are means (standard deviation [SD]) except for MVPA and television viewing which are geometric means (95% confidence interval [95% CI]).

† PAL = Physical Activity Level (*extremely inactive lifestyle*, PAL<1.40; *sedentary/lightly active lifestyle*, PAL 1.4–1.69; *moderately active lifestyle*, PAL 1.70–1.99, *very active lifestyle*, PAL≥2.0).

¥ Activity intensity based on MET value of activities: sedentary <1.5METS; light 1.5–≤3METS; moderate/vigorous >3METS.

a P-value based on linear regression with physical activity variables as the outcome, adjusted for age, sex and factory site and using robust standard errors to account for clustering (sib-pairs), log-transforming MVPA and television viewing and performing Wald tests on model parameters.

b Based on a subset of questions from the Standard of Living Index. Scores are based on tertiles.

c South includes the four southern states of Andhra Pradesh, Kerala, Karnataka and Tamil Nadu.

PA differed between migrant groups for all activities (p<0.001) except light intensity activity. Rural participants reported higher total activity, PAL (1.69 rural vs. 1.58 migrant vs. 1.59 urban) and time spent in MVPA (geometric mean; 142 min/day rural vs. 77 min/d migrant vs. 79 min/day urban) and sleep, but less time in sedentary behaviour (421 min/day rural vs. 491 min/day migrant vs. 508 min/day urban) and specifically television viewing (15 min/day rural vs. 50 min/day migrant vs. 55 min/day urban). There was strong evidence of differences in PA by sex. Men reported higher total activity, PAL and MVPA but less light activity, television viewing and sleep compared to women (*P*<0.001). Increasing age was associated with lower total activity, PAL, and time in light activity and sleep but higher sedentary behaviour (min/day). All PA variables varied across SEP except time spent in light activity (min/day). Increasing SEP was associated with decreased total activity, PAL, time in MVPA and sleep, but more time in sedentary behaviour (low SEP: 423 min/day vs. Medium SEP: 494 min/day vs. High SEP: 509 min/day), and television viewing. Participants from north India were more active, with higher total activity, PAL and time spent in light activity (min/day), compared to those from the south, who reported more television viewing and sleep (p<0.001). Sedentary behaviour and MVPA were not associated with geographic location.

### Physical activity by migrant status and sex

Table 3 shows PA patterns stratified by migrant status and sex. Women had lower levels of total activity, PAL and MVPA than men. Rural men reported in excess of 1 hour more MVPA, >1 hour less sedentary behaviour, >30 minutes less television viewing and >15 minutes more sleep daily than both migrant and urban men (P<0.001). With the exception of rural men, the majority of the population are sedentary or less active than sedentary. This proportion was greatest in migrant women (85%), urban women (80%), migrant men (80%) and urban men (75%). There was evidence of interactions between migrant status and sex on all PA variables (P<0.01).

**Table 3:** Distribution of physical activity variables by sex and migrant status (mean, [SD], geometric mean [95% CI] or percentage, [95% CI]).

	Men (n = 3,768)				Women (n = 2,679)			
	Rural	Migrant	Urban	p-value	Rural	Migrant	Urban	P-value
	(1,443)	(1,127)	(1,198)		(634)	(n = 967)	(1,078)	
**Total Activity (MET hr/day)**	41.3	38.5	38.2	<0.001	38.7	37.3	37.9(	<0.001
	(5.4)	(3.7)	(4.2)		(5.0)	(3.5)	3.6)	
**Activity Intensity (min/day)**								
Sedentary (<1.5 METS)	414	495	529	<0.001	437	486	485	<0.001
	(170)	(139)	(156)		(185)	(152)	(151)	
Light (1.5–3METS)	330	352	323	<0.001	410	421	424	0.388
	(119)	(118)	(126)		(121)	(132)	(131)	
Moderate/Vigorous (>3 METS)	195	126	120	<0.001	69	43	49	<0.001
	(185–205)	(119–133)	(114–126)		(62–78)	(40–47)	(45–54)	
**Physical Activity Level (PAL)**	1.72	1.60	1.59	<0.001	1.61	1.55	1.58	<0.001
	(0.22)	(0.15)	(0.17)		(0.21)	(0.15)	(0.15)	
**PAL grouped into lifestyle categories (%)**								
Extremely inactive lifestyle (PAL<1.40)	6.0	5.3	11.1		15.3	13.7	10.9	
	(4.8–7.3)	(4.0–6.6)	(9.3–12.9)		(12.5–18.2)	(11.5–15.8)	(9.0–12.7)	
Sedentary/Lightly active lifestyle (PAL 1.40–1.69)	42.4	74.2	64.0		56.0	70.8	69.5	
	(39.8–44.9)	(71.7–76.8)	(61.3–66.7)		(52.1–59.9)	(68.0–73.7)	(66.8–72.3)	
Moderately active lifestyle (PAL 1.70–1.99)	41.1	18.3	22.3		23.1	14.7	18.1	
	(38.5–43.6)	(16.0–20.6)	(19.9–24.6)		(19.8–26.4)	(12.5–16.9)	(15.8–20.4)	
Vigorously active lifestyle (PAL≥2.0)	10.5	2.1	2.6	<0.001	5.5	0.8	1.5	<0.001
	(9.0–12.1)	(1.3–3.0)	(1.7–3.5)		(3.8–7.3)	(0.3–1.4)	(0.8–2.2)	
**Duration TV Viewing (min/day)**	15	43	50	<0.001	17	59	62	<0.001
	(13–16)	(39–47)	(46–55)		(14–20)	(54–65)	(57–67)	
**Duration Sleep (min/day)**	444	429	429	<0.001	464	458	444	<0.001
	(63)	(58)	(64)		(65)	(61)	(63)	

Data presented in the table are means standard deviation (SD), except MVPA and TV viewing (geometric mean (95% Confidence Interval [95% CI]) and PAL categories which are percentages (95% CI).

PAL = Physical Activity Level (*extremely inactive lifestyle*, PAL<1.40; *sedentary/lightly active lifestyle*, PAL 1.4–1.69; *moderately active lifestyle*, PAL 1.70–1.99, *very active lifestyle*, PAL≥2.0).

P-value based on regression models with physical activity variables as the outcome, adjusting for age and factory site, and using robust standard errors to account for clustering, log-transforming moderate/vigorous activity and television viewing and performing Wald tests on model parameters.

### Multivariable Analyses of Total Activity (MET hr/day) and Migrant Status

Unadjusted analyses (accounting for clustering) showed that migrant men were less active than rural but more active than urban men (Table 4). Migrant men were less likely to be in the highest quartile of total activity compared to rural men (OR 0.25, 95% CI 0.19, 0.38), but more likely compared to urban men (1.19, 95% CI 0.93, 1.56). In both analyses the associations showed a non-linear trend across activity categories (p<0.001). After adjusting for SEP, education and occupation, differences in total activity (MET hr/day) between migrant and rural men persisted but attenuated (OR 0.53, 95% CI 0.37, 0.74). For migrant and urban men, differences attenuated after adjustment. For women unadjusted analyses showed that migrant women were less active than both rural and urban women (Table 5). Migrant women were less likely to be in the highest category of total activity than the lowest category compared to rural women (OR 0.56, 95% CI 0.43, 0.73; p-trend [non-linear <0.001]) and urban women (OR 0.61, 95% CI 0.50, 0.79; p-trend [linear <0.001]). After adjusting for SEP, occupation and education, these associations were fully attenuated.

**Table 4:** Crude and adjusted odds ratios (OR) and 95% confidence intervals (CI) for migrant status (migrant vs. rural and migrant vs. urban) by Total Activity (MET hr/day) for men within the IMS.

	Men				Men			
	Migrant vs. Rural (n = 2,570)				Migrant vs. Urban (2,325)			
	Migrant/Rural (n)	Unadjusted (accounting for clustering)	Adjusted for age and site and accounting for clustering	Adjusted for age, site, occupation, SES, education and accounting for clustering	Migrant/Urban (n)	Unadjusted (accounting for clustering)	Adjusted for age and site and accounting for clustering	Adjusted for age, site, occupation, education and accounting for clustering
**Total Activity (MET hr/day)**								
Quartile 1 (lowest)	273/236	1.00	1.00	1.00	273/397	1.00	1.00	1.00
Quartile 2	275/170	1.40 (1.08–1.81)	1.45(1.11–1.90)	1.33(0.94–1.87)	275/246	1.63(1.30–2.05)	1.67(1.31–2.13)	1.39(1.08–1.79)
Quartile 3	410/418	0.85 (0.68–1.05)	0.88(0.70–1.11)	1.04(0.77–1.42)	410/305	1.70(1.38–2.10)	1.71(1.37–2.13)	1.41(1.12–1.79)
Quartile 4 (highest)	169/618	0.24(0.19–0.30)	0.24(0.19–0.38)	0.53(0.37–0.74)	169/205	1.19(0.93–1.56)	1.18(0.89–1.55)	0.99(0.74–1.34)
p-association		<0.001	<0.001	<0.001		<0.001	<0.001	<0.001
P-trend (non-linear)		<0.001	<0.001	<0.001		<0.001	<0.001	<0.001

Data presented are odds ratio (OR) and 95% confidence intervals (95% CI). OR presented are odds of being a migrant compared to a rural or urban participant across categories of Total Activity (MET hr/day).

P-trend (non-linear) from logistic regression using migrant status as the outcome and categories of Total Activity (MET hr/day) as the exposure variable, adjusting as specified in models above.

**Table 5:** Crude and adjusted odds ratios (OR) and 95% confidence intervals (CI) for migrant status (migrant vs. rural and migrant vs. urban) by Total Activity (MET hr/day) for women within the IMS.

	Women				Women			
	Migrant vs. Rural (n = 1,601)				Migrant vs. Urban (n = 2,045)			
	Migrant/Rural (n)	Unadjusted (accounting for clustering)	Adjusted for age and site and accounting for clustering	Adjusted for age, site, occupation, SES, education and accounting for clustering	Migrant/Urban (n)	Unadjusted (accounting for clustering)	Adjusted for age and site and accounting for clustering	Adjusted for age, site, occupation, education and accounting for clustering
**Total Activity (MET hr/day)**								
Quartile 1 (lowest)	264/166	1.00	1.00	1.00	264/228	1.00	1.00	1.00
Quartile 2	237/110	1.35(1.01–1.81)	1.16(0.86–1.57)	1.06(0.72–1.56)	237/238	0.86(0.66–1.11)	1.02(0.79–1.33)	1.04(0.78–1.40)
Quartile 3	272/141	1.21(0.92–1.59)	1.02(0.76–1.36)	1.08(0.75–1.56)	272/338	0.69(0.55–0.89)	0.86(0.67–1.11)	0.96(0.72–1.28)
Quartile 4 (highest)	194/217	0.56(0.43–0.73)	0.49(0.37–0.64)	0.88(0.61–1.25)	194/274	0.61(0.50–0.79)	0.72(0.55–0.95)	0.89(0.65–1.21)
p-association		<0.001	0.041	0.669		<0.001	0.041	0.299
P-trend (non-linear)		0.013	0.025	0.309		0.114	0.070	0.375

Data presented are odds ratio (OR) and 95% confidence intervals (95% CI). OR presented are odds of being a migrant compared to a rural or urban participant across categories of Total Activity (MET hr/day).

P-trend (non-linear) from logistic regression using migrant status as the outcome and categories of Total Activity (MET hr/day) as the exposure variable, adjusting as specified in models above.

## Discussion

A mean PAL of 1.62 (SD, 0.19) for the population as a whole equates to a sedentary/lightly active lifestyle and is lower than values from studies from within the USA [33] and UK [34]. Time (min/day) spent in sedentary and light activities was high across all groups, indicating that the physical activity transition [22] is already underway, particularly amongst urban and migrant participants. Conversely however, our findings of 95 min/day (95% CI 92, 98) spent in MVPA for the population as a whole suggests that the population is meeting PA guidelines and that sedentary activity or alternative factors may play a bigger role in determining PAL. Migrants were less active than rural participants with activity patterns more similar to urban participants. Socio-demographic factors (age, factory site, education, SEP and occupation) explained variation in PA between migrant groups among women whilst in men these differences remained but attenuated. The similarity between migrants and urban non-migrants further suggests that structural rather than early life learnt behaviour may be the more important determinants of PA within this population.

### Results in context of other studies

Previous research on PA within India has tended to focus on specific geographic locations or sub-groups, using different methods to quantify PA and has not attempted to examine differences between migrants and other groups [35], [36], [37], [38]. The higher levels of activity seen amongst rural participants compared to migrant and urban residents within this study are however concordant with previously published migration study data from other LMIC [39].

The associations of PA with age observed here are consistent with previous research in southern India [40]. Higher levels of PA in men is well established within India [40] and other LMIC [41], [42], and was expected due to the higher proportion involved in manual occupations (54% men; 10% women). Lower activity levels in women may also be attributable to greater involvement in domestic chores [35], [36], (77% of women and 1% of men reported housework as their main occupation), activities which tend to be of a lower activity intensity than paid manual occupations.

Activity levels reported for both urban and rural men were lower than those reported in a study from Cameroon although comparable for women [41], who also showed consistent levels with a previous study from rural India [38]. Lower levels of total activity in urban areas have been noted in other studies in both north [2] and south India where PA was negatively associated with level of urbanisation for both men and women [43].

The finding that higher SEP was associated with lower PA participation was also concordant with published data in adults and adolescents in LMIC and HIC settings [42] and reflects the non-manual/professional occupations in the higher SEP groups (13%, 24% and 40% non-manual/professional occupation in the low, middle and high SES groups respectively).

The design of the IMS produced strengths and limitations: The broad, diverse and detailed nature of the IMS containing clinical, anthropometric and lifestyle data on 6,447 participants across migrant and socio-demographic groups (sex, age, SES, north/south) in four regions of India are one of its main strengths. The IMS-PAQ, which has been previously validated [29], allows participants to report on up to 42 separate activities, providing rich and diverse data on habitual activity recalled over the previous month.

Use of PA information based on self-reported participation over ‘last one-month’ may result in recall or reporting bias. It is possible that measurement errors would be differential by migration status as the range of possible PA differs between urban and rural settings. The validation of the PA instrument [29], the repeated training given to interviewers, and the payment of costs associated with participation in the study mitigated against some of these possible sources of bias.

Recruitment for this study focussed on workers and their associated siblings and spouses from four factory sites across India which, as previously reported [18], resulted in a wealthier, more educated and less illiterate population than the urban or rural national average. The response rate of ∼50% may have given rise to the additional issue of selection bias and as such, the true prevalence of PA may be inaccurately estimated in this sample. The results presented are not generalisable to the Indian population as a whole but may represent the segment of the Indian population that is in the vanguard of social change and is experiencing urbanisation and changes in lifestyle.

### Implications of the study findings

Quantifying PA patterns amongst rural, migrant and urban groups in India will increase understanding of the potential health burden the country may face in the future. Predictions about India's population growth and urbanisation, estimate that by 2025 approximately 605–613 million people may be urbanised [2], [4], a process in part driven by rural-urban migration. This study has identified that total activity levels and time spent in MVPA are significantly lower amongst urban residents, older participants, women and those in higher SEP, whilst television viewing and sedentary behaviour levels are markedly higher. The epidemic of obesity and type II diabetes in India is likely to worsen as urbanisation increases, economic growth continues to raise SEP [3], [44], and underlying and marked reductions in PA occur.

Our findings indicate that in this diverse study population the majority of people are leading sedentary lives (PAL 1.62) reflecting their age, employment status, rapid urbanisation and SEP. To move this population to moderately active lifestyles (PAL 1.70) would require an increase in the mean PAL of 0.08. Approximately 60 min/day brisk walking (≥6<7.5 km/per hr) would raise PAL by 0.2, and 60 min/day of jogging (9 km/per hr) would raise PAL by 0.4 [33]. The urban environment in most Indian cities is ‘automobile-oriented’ [45] which makes achievement of even these modest increases in PA unlikely without a dramatic reversal of current planning policies that favour motorised transportation to walking or cycling [46]. These low PAL findings are however discordant with the high levels of MVPA reported amongst this population. Whilst over-reporting may partly explain such variation, another possible reason could be ethnic variations in BMR, the key denominator for calculating PAL. Previous research suggests that BMR in Indians could be 10% lower than values produced from currently accepted formula [47], [48], with further variation by sex and SEP [49]. Reducing BMR accordingly within our population results in a mean PAL of 1.81, equivalent to a moderately active lifestyle, although further widening the disparity in PAL by SEP.

### Conclusion

Our findings suggest that PA levels are low among the growing more advantaged populations in urban areas of India. The high amounts of time spent in sedentary and light activities, especially television viewing, indicate that the PA transition is already underway in rural, migrant and urban groups. There is now an urgent need to collect data on BMR from both rural and urban India, and to investigate how to design better urban environments and develop appropriate and effective public health interventions to increase PA and thereby control the current obesity and diabetes epidemics.
